# Mediterranean style diet is associated with low risk of new-onset diabetes after renal transplantation

**DOI:** 10.1136/bmjdrc-2016-000283

**Published:** 2017-01-13

**Authors:** Maryse C J Osté, Eva Corpeleijn, Gerjan J Navis, Charlotte A Keyzer, Sabita S Soedamah-Muthu, Else van den Berg, Douwe Postmus, Martin H de Borst, Daan Kromhout, Stephan J L Bakker

**Affiliations:** 1 Department of Internal Medicine, Division of Nephrology, University of Groningen, University Medical Center Groningen, Groningen, The Netherlands; 2 Department of Epidemiology, University of Groningen, University Medical Center Groningen, Groningen, The Netherlands; 3 Department of Human Nutrition, University of Wageningen, Wageningen, The Netherlands

**Keywords:** Renal Transplantation, Dietary Patterns, Post-Transplant Diabetes, Mortality

## Abstract

**Objective:**

The incidence of new-onset diabetes after transplantation (NODAT) and premature mortality is high in renal transplant recipients (RTR). We hypothesized that a Mediterranean Style diet protects against NODAT and premature mortality in RTR.

**Research design and methods:**

A prospective cohort study of adult RTR with a functioning graft for >1 year. Dietary intake was assessed with a 177-item validated food frequency questionnaire. Patients were divided based on a 9-point Mediterranean Style Diet Score (MDS): low MDS (0–4 points) versus high MDS (5–9 points). A total of 468 RTR were eligible for analyses. Logistic multivariable regression analyses were used to study the association of MDS with NODAT and Cox multivariable regression models for the association with all-cause mortality.

**Results:**

Mean±SD age was 51.3±13.2 years and 56.6% were men. About 50% of the patients had a high MDS. During median follow-up of 4.0 (IQR, 0.4–5.4) years, 22 (5%) RTR developed NODAT and 50 (11%) died. High MDS was significantly associated with both a lower risk of NODAT (HR=0.23; 95% CI 0.09 to 0.64; p=0.004) and all-cause mortality (HR=0.51; 95% CI 0.29 to 0.89, p=0.02) compared to low MDS, independent of age and sex. Adjustment for other potential confounders, including total energy intake, physical activity and smoking status, did not materially change the results of the analyses.

**Conclusions:**

Dietary habits leading to high MDS were associated with lower risk of NODAT. These results suggest that healthy dietary habits are of paramount importance for RTR.

**Key messages**

- Renal transplant recipients are at high risk for development of new-onset diabetes after transplantation and premature mortality.
- Dietary habits consistent with a Mediterranean style diet are associated with lower risk of development of new-onset diabetes after transplantation and premature mortality in renal transplant recipients.
- These lower risks were independent of potential confounders, including age, sex, physical activity and smoking behavior.

## Introduction

The prevalence of patients with end-stage renal disease for which patients require chronic dialysis or renal transplantation, also called renal replacement therapy, is increasing at a rate of 7% per year.^1^ Renal transplantation improves quality of life and increases survival, compared to dialysis treatment.^2^ ^3^ However, the results are not impressive, since a lot of factors impair survival after renal transplantation. One of these factors is new-onset diabetes after transplantation (NODAT), which is caused by the combination of insulin resistance and deficient insulin production.^4^ The incidence of NODAT is high and affects ∼20% of renal transplant recipients (RTR).^5^ However, a systemic review of Montori *et al*^6^ concluded that the incidence of NODAT varied widely from 2% to 50% in the first post-transplant year, due to inconsistencies regarding the definition of NODAT. NODAT is an important risk factor for premature mortality in RTR.^7^ ^8^ This can partly be explained by the fact that NODAT contributes to a high cardiovascular risk, infectious complications and impaired graft survival in RTR.^9–11^

Patients with end-stage renal disease are often advised to consume a low protein, low potassium, low sodium diet, which is also often restricted in fluid intake, causing a diet high in energy-rich drinks, carbohydrates and fats to get enough energy.^12^ This diet prevents excessive generation of urea and the occurrence of hyperphosphatemia, hyperkalemia and hypertension. However, a low-protein diet gives little satiety,^13^ and adherence to this dietary pattern may induce problems once combined with the stimulation of appetite. This may be a consequence of improved renal function after renal transplantation and as a side effect of the corticosteroid treatment that is part of the immunosuppressive regimen after renal transplantation.^14^ Furthermore, RTR have low levels of physical activity and gain fat mass after transplantation, resulting in obesity in 26% of the participants,^12^ an independent risk factor for the development of NODAT.^9^ To reduce the incidence of NODAT and improve overall transplant success, more attention should be paid to lifestyle modification.

Meta-analyses showed that adherence to the Mediterranean diet was associated with a lower risk of diabetes.^15^ ^16^ Furthermore, Estruch *et al*^17^ showed in the PREDIMED trial that two components of the Mediterranean diet (additional amount of extra virgin olive oil and nuts) reduced the incidence of cardiovascular events among high-risk patients. In the present study, we aimed to investigate the association of Mediterranean Style diet with the incidence of NODAT and all-cause mortality in RTR that has not yet been examined. We hypothesized that a Mediterranean Style diet is associated with a lower risk of NODAT and all-cause mortality in RTR.

## Research design and methods

### Study design and population

The study design of this research project is a large single-center prospective cohort of RTR. All adult RTR (≥18 years) with a functioning graft for at least 1 year who visited the outpatient clinic in the University Medical Center Groningen between November 2008 and May 2011 were invited to participate. Patients were only included if this visit was at least 1 year after transplantation. All patients had sufficient knowledge of the Dutch language and according to their patient files and no history of drug or alcohol addiction. In total 707 (86.5%) of the initially 817 invited RTR signed a written informed consent. RTR with missing dietary data (n=81), diabetes mellitus (DM) at baseline (n=152) or who underwent combined pancreas-kidney transplantation (n=6) were excluded, leaving 468 RTR eligible for analyses. This research project was approved by the institutional review board (METc 2008/186), which adhered to the Declaration of Helsinki.

### Data collection and clinical end points

Baseline data were collected during a morning visit to the outpatient clinic as described previously.^18^ ^19^ Body weight and height were measured while participants wore indoor clothing without shoes. Waist and hip circumference were measured. Both systolic blood pressure and diastolic blood pressure and also heart rate were measured using a semiautomatic device (Dinamap1846; Critikon, Tampa, Florida, USA). They were measured every minute for 15 min in a half-sitting position to prevent white coat hypertension. Information about daily physical activity was derived using the *S*hort *QU*estionnaire to *AS*sess *H*ealth-enhancing physical activity (SQUASH) score in time multiplied by intensity.^20^ Information about smoking behavior was obtained by using a questionnaire. Medication use was acquired from patient records. Furthermore, fasting blood samples were collected and patients were also asked to complete 24 hour urine collection. All RTR were informed to discard their morning urine specimen and then collect their urine for the next 24 hours, including the next morning's first specimen of the day of their visit. Creatinine clearance was based on 24 hour urinary creatinine and serum creatinine. Estimated glomerular filtration rate was calculated using the serum creatinine-based Chronic Kidney Disease Epidemiology Collaboration equation.^21^

A semiquantitative food frequency questionnaire (FFQ) was used to collect information on dietary intake at baseline during the past month. The FFQ was developed at Wageningen University^22^ and consisted of 177 items. Patients filled out the self-administered FFQ at home. Frequency was recorded in times per day, week or month for each item. Expression of number of servings was in either natural units such as a slice of bread or an apple, or in household measures, for example, a cup or a teaspoon. Subsequently, all dietary data were converted into total energy and nutrient intake per day using the Dutch Food Composition Table (NEVO 2006). The FFQ was validated by comparing the protein intake of the FFQ with the protein intake calculated by the Maroni Equation, using urinary urea excretion values.^19^ Protein intake was similar to the estimates derived from the FFQ.^23^

The degree to which the consumed diet resembled the traditional Mediterranean diet was calculated according to a nine-point Mediterranean Diet Score (MDS) of Trichopoulou *et al*.^24^ The MDS includes nine food groups: ratio of monounsaturated:saturated fatty acids, intake of legumes, cereals, vegetables, fruit, fish, dairy products, meat products and alcohol.^9^ ^14^ Food items of the FFQ were divided over these nine food groups (see online supplementary table S1). For each food group, the sex-specific median in grams per day was used as cut-off point for making this division, except for fish and alcohol. Patients received a score of 1 for each of the putative protective components (ratio of monounsaturated:saturated fatty acids, legumes, cereals, vegetables, fruit) if their intake was above the median. The traditional Mediterranean diet was low in dairy and meat products. Therefore, an intake below the median for these food groups was scored 1 and for an intake above the median 1. For the fish component participants received a score of 1 if they consumed more than 5 g/day and a score of 0 if they consumed <5 g/day (<once a month). Alcohol users received a score of 1 and non-users received a score of 0. Moderate alcohol intake is associated with low prevalence of NODAT and lower risk for mortality in RTR, when compared to abstainers.^25^ The MDS varies between 0 (lowest adherence) and 9 (highest adherence). Subsequently, all patients were divided into two groups based on the frequency distribution of the MDS: group 1 (MDS 0–4) and group 2 (MDS 5–9). We dichotomized data because of the small number of events.

10.1136/bmjdrc-2016-000283.supp1 supplementary table Overview of the food items.

In this study primary outcome measurements are the incidence of NODAT and all-cause mortality. Care-based data about the incidence of NODAT and mortality after baseline were retrieved from patient files of all RTR until 1 April 2014. NODAT was defined as a fasting plasma glucose level ≥7.0 mmol/L and/or use of oral hypoglycemic agents or insulin therapy for ≥30 consecutive days.^26^ Patients developed NODAT when they had one or more of the following conditions; the patient was diagnosed with DM, used antidiabetics (oral hypoglycemic agents or insulin therapy) and/or had a fasting plasma glucose level ≥7.0 mmol/L or non-fasting glucose level ≥11.1 mmol/L. The diagnosis of NODAT was made in outpatient and clinical routine. Patients were tested repeatedly before the diagnosis of NODAT was made and treatment was started. No participants were lost to follow-up.

Data on use of diuretics and/or β blockers, use of ACE-inhibitors or angiotensin II receptor blocker, use of statins, prevalence of polycystic kidney disease and nephrosclerosis, previous viral infections (hepatitis C and cytomegalovirus), cumulative dose of steroids before inclusion, but during follow-up, incidence of acute rejection episodes during follow-up and used of mTOR inhibitors at baseline and during follow-up were retrieved from individual patient files. Cumulative dose of prednisolone was calculated as the sum of maintenance dose of prednisolone until inclusion and the dose of prednisolone or methylprednisolone required for treatment of acute rejection (a conversion factor of 1.25 was used to convert methylprednisolone dose to dose of prednisolone).

### Statistical analyses

Variable distribution was tested with histograms and probability (Q-Q) plots. For descriptive statistics, data are presented as mean and SD when normally distributed, median and IQR when skewed distributed and number and percentage in case of categorical data. Differences between the two MDS groups to test for potential confounders were tested by an unpaired t-test for continuous variables with a normal distribution, Mann-Whitney U test for continuous variables with a skewed distribution and by means of a χ^2^ test for categorical variables. All statistical analyses were performed using IBM Statistics SPSS V.22.0. For all statistical tests a statistical significance level of p≤0.05 (two-tailed) was used. GraphPad Prism 5 was used to generate the figures.

Primary analyses concerned Kaplan-Meier curves of the incidence of NODAT and all-cause mortality. For NODAT multivariable logistic regression models were used because the exact dates when patients developed an event were not exactly known. For multivariable Cox regression models patients were censored at the date of last follow-up or death. Owing to the small number of NODAT and all-cause mortality the models were first adjusted for age and sex and additionally for more than two potential confounders.^27^ Models were checked for fulfillment of the assumptions for logistic regression and Cox regression, including the proportional hazards assumption. The assumptions were met.

## Results

**Figure 1:**
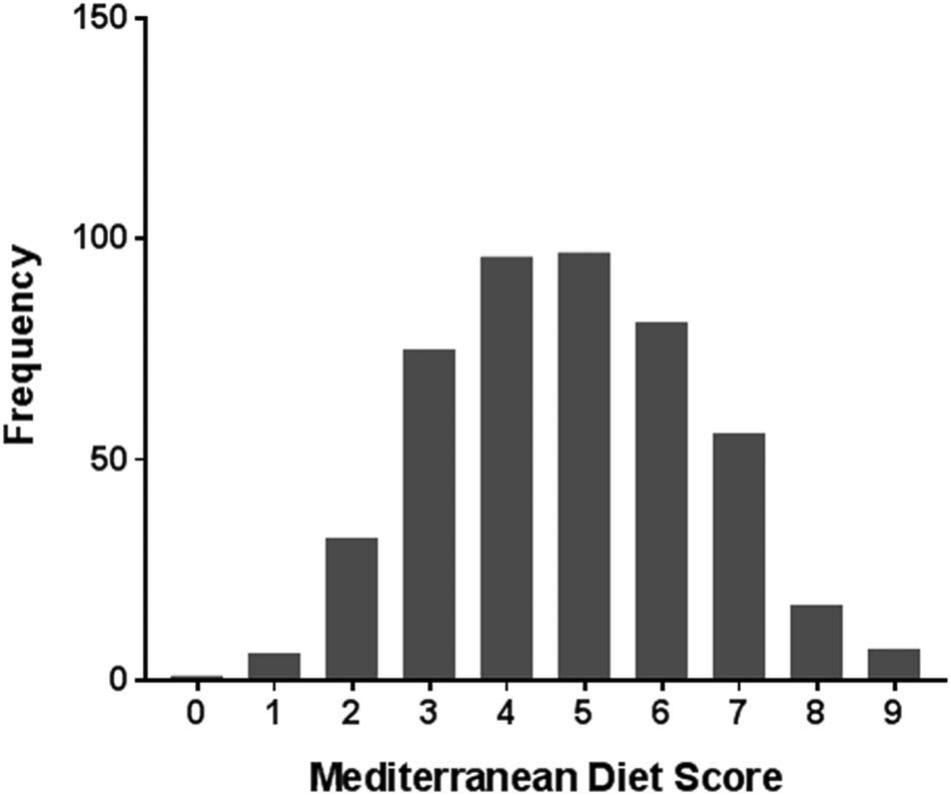
Frequency distribution of the Mediterranean Diet Score (MDS) in the overall RTR population (468 participants). RTR, renal transplant recipients.

In total 468 RTR (56.6% men) were included with a mean±SD age of 51.3±13.2 years. The frequency distribution of the MDS in these 468 RTR is presented in figure 1. The MDS varied between 0 (lowest adherence) and 9 (highest adherence), with a mean score of 4.8±1.7 and 54% of the patients had a high score (>4). Baseline characteristics of the overall RTR population and according to high versus low MDS are shown in table 1. Age and physical activity differed significantly between the groups. Patients with a high MDS were older, had a higher physical activity score, lower fasting triglycerides and higher high-density lipoprotein (HDL)-cholesterol concentrations compared to patients with a low MDS. The percentage of smokers and total energy intake did not differ. A borderline statistical significance was found for pre-emptive transplantation, cold ischemia time and use of tacrolimus.

**Table 1:** Baseline characteristics of the overall RTR population and according to groups based on the MDS

	Overall RTR (n=468)	Group 1 MDS 0–4 (n=217)	Group 2 MDS 5–9 (n=251)	p Value
Demographics				
Age, years	51.3±13.2	49.9±13.9	52.5±12.4	0.03
Male gender, n (%)	265 (56.6)	124 (57.1)	141 (56.2)	0.83
Smoking behavior (current smoker), n (%)	60 (12.8)	29 (13.4)	31 (12.4)	0.64
Total energy intake, kcal/day	2199±656	2168±696	2225±619	0.35
Physical activity score (time×intensity)	5605 (2885–8647)	5060 (2070–8385)	6000 (3480–8700)	0.03
Weight, kg	78.9±15.8	78.1±16.2	79.6±15.5	0.31
Body composition				
Height, cm	173.9±9.7	173.4±10.6	174.3±9.0	0.30
BMI, kg/m^2^	26.0±4.5	25.9±4.6	26.1±4.5	0.60
Waist circumference, cm				
Men	99.1±12.1	98.7±12.2	99.5±12.1	0.98
Women	93.0±15.8	92.6±15.3	93.4±16.3	0.68
Circulation				
Heart rate, bpm	67.6±12.0	67.8±12.3	67.4±11.8	0.72
SBP, mm Hg	135.3±17.0	135.7±16.3	135.0±17.7	0.68
DBP, mm Hg	83.0±11.0	83.3±11.1	82.8±11.0	0.61
Renal function				
eGFR, mL/min per 1.73 m^2^	53.1±20.2	52.3±21.6	53.7±18.8	0.45
Laboratory parameters				
Triglycerides, mmol/L	1.6 (1.2–2.1)	1.7 (1.2–2.3)	1.5 (1.1–2.0)	0.04
HDL cholesterol, mmol/L	1.4±0.5	1.4±0.4	1.5±0.5	0.001
Fasting glucose, mmol/L	5.1 (4.7–5.5)	5.1 (4.7–5.5)	5.0 (4.7–5.5)	0.51
Hepatitis C virus, n (%)	6 (1.3)	3 (1.4)	3 (1.2)	0.86
Cytomegalovirus, n (%)				
Primary infection	99 (21.2)	48 (22.1)	51 (20.3)	0.63
Reactivation	80 (17.1)	38 (17.5)	42 (16.7)	0.69
Primary renal disease				
Polycystic kidney disease, n (%)	103 (22.0)	47 (21.7)	56 (22.3)	0.87
Nephrosclerosis, n (%)	139 (29.7)	67 (30.9)	72 (28.7)	0.61
Transplant characteristics				
Transplant vintage, years	5.6 (2.1–12.3)	5.2 (2.2–12.3)	5.8 (1.8–12.3)	0.80
Living donor, n (%)	168 (35.9)	70 (32.3)	98 (39.0)	0.20
Pre-emptive transplant, n (%)	84 (17.9)	32 (14.7)	52 (20.7)	0.09
Dialysis duration, months	37.0 (16.0–60.0)	46.0 (15.0–63.0)	32.0 (17.0–56.0)	0.30
Age donor, years	43.0±15.5	42.6±15.0	43.4±15.9	0.61
Cold ischemia time, hours	14.0 (3.0–21.0)	16.0 (3.0–21.0)	12.0 (3.0–21.0)	0.06
Warm ischemia time, minutes	40 (33–50)	42 (33–51)	39 (34–48)	0.25
Acute rejection, n (%)	114 (24.4)	49 (22.6)	65 (25.9)	0.41
Medication				
Cyclosporine, n (%)	178 (38.0)	82 (37.8)	96 (38.2)	0.92
Tacrolimus, n (%)	79 (16.9)	44 (20.3)	35 (13.9)	0.07
mTOR inhibitor, n (%)	5 (1.1)	2 (0.9)	3 (1.2)	0.76
Prednisolone dose, mg	10.0 (7.5–10.0)	10.0 (7.5–10.0)	10.0 (7.5–10.0)	0.70
Cumulative prednisolone dose, g	18.3 (7.4–38.1)	18.3 (7.8–36.6)	18.2 (7.4–40.4)	0.86
Diuretics, n (%)	158 (33.8)	76 (35.0)	82 (32.7)	0.59
β blocker, n (%)	284 (60.7)	126 (58.1)	158 (62.9)	0.28
ACE inhibitor, n (%)	158 (33.8)	73 (33.6)	85 (33.9)	0.96
Angiotensin II receptor blocker, n (%)	71 (15.2)	39 (18.0)	32 (12.7)	0.12
Statins, n (%)	232 (49.6)	100 (46.1)	132 (52.6)	0.15

Data are represented as mean±SD, median (IQR) or n (%). Differences were tested by t-test or Mann-Whitney U test for continuous variables and with χ^2^ test for categorical variables.

BMI, body mass index; DBP, diastolic blood pressure; eGFR, estimated glomerular filtration rate; HDL, high-density lipoprotein; MDS, Mediterranean Diet Score; RTR, renal transplant recipients; SBP, systolic blood pressure.

Median time between baseline and transplantation was 5.6 (IQR, 2.1–12.3) years. During a median follow-up of 4.0 (IQR, 0.4–5.4) years from baseline, 22 (5%) patients developed NODAT (17 RTR in low MDS group, 5 RTR in high MDS group) and 50 (11%) patients died (29 RTR in low MDS group, 21 RTR in high MDS group). In the low MDS group, 15 (88%) of the RTR that developed NODAT required treatment with hypoglycemic agents or insulin and in the high MDS group, 5 (100%) of the RTR that developed NODAT required treatment with hypoglycemic agents or insulin. Median intake for the nine food groups of the MDS for both men and women are shown in table 2. In the low MDS group men had a higher intake of cereals and alcohol, whereas women had a higher intake of fruit. In the high MDS group men had a higher intake of cereals, whereas women had a high intake of fruit and vegetables. Intake of other food groups is approximately equal between male and female RTR. The high MDS group had a higher intake of legumes, cereals, vegetables, fruit, fish and alcohol compared to the low MDS group.

**Table 2:** Median intake of the components of the Mediterranean Diet Score

	Group 1 (0–4) Median (IQR)		Group 2 (5–9) Median (IQR)	
	Men	Women	Men	Women
Ratio monounsaturated: saturated fatty acids	0.9 (0.8–1.0)	0.9 (0.8–1.0)	1.0 (0.9–1.1)	1.0 (0.9–1.1)
Legumes, nuts and soy products (g/day)	29 (16–39)	28 (18–40)	52 (38–72)	44 (32–71)
Cereals (g/day)	176 (128–240)	134 (107–178)	210 (170–257)	175 (141–206)
Fruit (g/day)	77 (34–137)	105 (57–211)	135 (81–234)	211 (97–249)
Vegetables (g/day)	57 (32–77)	64 (48–87)	100 (74–140)	124 (92–153)
Meat products (g/day)	109 (82–128)	94 (77–116)	90 (70–115)	79 (58–99)
Dairy products (g/day)	357 (234–511)	399 (253–492)	330 (211–481)	369 (217–507)
Fish (%)	34	34	57	67
Alcohol (%)	67	84	48	80

**Figure 2:**
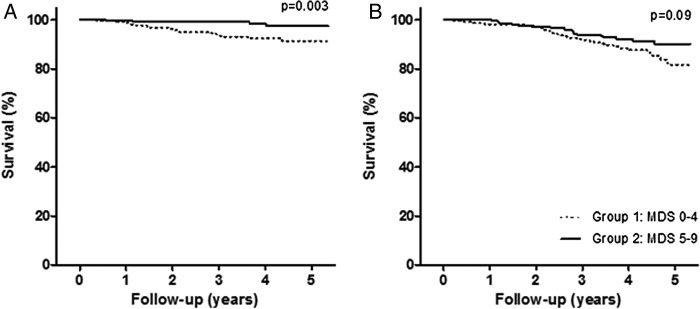
Kaplan-Meier survival curves. Probability of survival for NODAT (A) and all-cause mortality (B) for both group 1 and group 2. NODAT, new-onset diabetes after transplantation.

The Kaplan-Meier survival curves for the associations of the MDS with NODAT (p=0.003, log rank test) and all-cause mortality (p=0.09, log rank test) are shown in figure 2. RTR with MDS scores ≥5 points were significantly associated with a lower risk of NODAT (OR: 0.23; 95% CI 0.09 to 0.64; p=0.004) and all-cause mortality (hazards ratio (HR): 0.51; 95% CI 0.29 to 0.89, p=0.02), adjusted for age and sex (tables 3 and 4). The results of multivariable analyses, in which we additionally adjusted for use of medication, pre-emptive transplantation and cold ischemia time, total energy intake, smoking behavior and physical activity, fasting triglycerides and HDL-cholesterol concentrations and time between transplantation and baseline, did not materially change the results of the analyses adjusted for age and sex (tables 3 and 4).

**Table 3:** Multiple logistic regression analysis

	Group 1 (0–4)	Group 2 (5–9)	
	17 (7.8%)	5 (2.0%)	
Number of events		OR (95% CI)	p Value
Model 1	1.00 (ref)	0.24 (0.09 to 0.66)	0.006
Model 2	1.00 (ref)	0.23 (0.08 to 0.63)	0.004
Model 3	1.00 (ref)	0.22 (0.08 to 0.62)	0.004
Model 4	1.00 (ref)	0.24 (0.08 to 0.69)	0.008
Model 5	1.00 (ref)	0.23 (0.08 to 0.63)	0.004
Model 6	1.00 (ref)	0.23 (0.08 to 0.65)	0.005
Model 7	1.00 (ref)	0.18 (0.06 to 0.54)	0.002
Model 8	1.00 (ref)	0.23 (0.08 to 0.63)	0.004

The Mediterranean diet is associated with a lower risk to develop NODAT.

Model 1, crude.

Model 2, adjustment for age and sex.

Model 3, model 2+adjustment for cyclosporine, tacrolimus and prednisolone dose.

Model 4, model 2+adjustment for pre-emptive transplantation and cold ischemia time.

Model 5, model 2+adjustment for total energy intake.

Model 6, model 2+adjustment for smoking and physical activity.

Model 7, model 2+adjustment for triglycerides and HDL-cholesterol concentrations.

Model 8, model 2+adjustment for time between transplantation and baseline.

HDL, high-density lipoprotein; NODAT, new-onset diabetes after transplantation.

**Table 4:** Cox Regression analysis

	Group 1 (0–4)	Group 2 (5–9)	
	29 (13.4%)	21 (8.4%)	
Number of events		HR (95% CI)	p Value
Model 1	1.00 (ref)	0.62 (0.35 to 1.09)	0.09
Model 2	1.00 (ref)	0.51 (0.29 to 0.89)	0.02
Model 3	1.00 (ref)	0.52 (0.29 to 0.92)	0.03
Model 4	1.00 (ref)	0.52 (0.27 to 0.99)	0.05
Model 5	1.00 (ref)	0.51 (0.29 to 0.89)	0.02
Model 6	1.00 (ref)	0.57 (0.22 to 1.03)	0.06
Model 7	1.00 (ref)	0.57 (0.32 to 1.02)	0.06
Model 8	1.00 (ref)	0.50 (0.29 to 0.89)	0.02

The Mediterranean diet is associated with a lower risk of mortality during follow-up.

Model 1, crude.

Model 2, adjustment for age and sex.

Model 3, model 2+adjustment for cyclosporine, tacrolimus and prednisolone dose.

Model 4, model 2+adjustment for pre-emptive transplantation and cold ischemia time.

Model 5, model 2+adjustment for total energy intake.

Model 6, model 2+adjustment for smoking and physical activity.

Model 7, model 2+adjustment for triglycerides and HDL-cholesterol concentrations.

Model 8, model 2+adjustment for time between transplantation and baseline.

HDL, high-density lipoprotein.

## Conclusions

About 50% of the RTR had either a low or a high MDS. Patients with a high MDS had a four times lower risk of NODAT and a two times lower risk of all-cause mortality. These results suggest that a healthy diet is of paramount importance for patients who receive a new kidney.

Previous studies on diet and chronic diseases often focused on single nutrients. However, food-based dietary patterns take into account complex interactions between food items and are easier to interpret. Another advantage of foods and dietary patterns is that they can directly be transplanted into dietary recommendations to be used in clinical practice.^28^ ^29^

A meta-analysis of population-based prospective cohort studies showed that greater adherence to the Mediterranean Style diet was associated with a 20% lower all-cause mortality^30^ and 20% lower cardiovascular risk.^31^ A meta-analysis of the adherence to the Mediterranean Style diet showed a 25% lower incidence of diabetes mellitus among prospective cohort studies.^15^

To the best of our knowledge, this is the first study to investigate the association between a Mediterranean Style diet and risk of NODAT and all-cause mortality in RTR. In line with our results on NODAT another study showed an association between adherence to a Mediterranean Style diet and a lower incidence of metabolic syndrome after transplantation.^32^ Prospective cohort studies among patients with cardiovascular disease showed that a Mediterranean Style diet was associated with a lower risk of all-cause mortality.^33–35^ These results suggest that a Mediterranean Style diet is associated with a lower mortality risk in patients with renal transplant and also in patients with cardiovascular disease. This shows the great potential of a healthy diet in secondary prevention.

There are multiple mechanisms that might explain the protective effect of a Mediterranean Style diet on the development of NODAT and mortality in RTR. It is well known that insulin resistance and pancreatic β cell dysfunction are two fundamental features that play an important role in the development of type 2 DM.^36^ High adherence to the Mediterranean Style diet is associated with a higher intake of antioxidants, dietary fiber, magnesium and unsaturated fatty acids.^36^ The Mediterranean diet may prevent cardiometabolic diseases through several pathways. First of all, prolonged oxidative stress contributes to the development of insulin resistance and pancreatic β-cell dysfunction.^37^ A Mediterranean Style diet might have a protective effect on oxidative stress and antioxidant defense, since this dietary pattern is characterized by high intake of fruit and vegetables.^38^ Second, the high intake of dietary fiber might reduce plasma insulin levels and have an advantageous effect on glucose metabolism.^36^ Third, magnesium might play an important role in preventing type 2 DM.^36^ Previous studies showed that high intake of magnesium is associated with a lower risk of developing type 2 diabetes.^39^ ^40^

Also fatty acids could play a role in the prevention of cardiometabolic diseases. A high ratio of monounsaturated: saturated fatty acids improves insulin sensitivity.^41^ A high intake of monounsaturated fatty acids benefits glycemic control, since it stimulates the secretion of glucagon-like peptide-1 (GLP-1), an antidiabetic hormone.^42^ GLP-1 activates the GLP-1 receptor in the pancreatic islets, which leads to an increase in secretion of insulin and inhibition of glucagon.^43^ Furthermore, GLP-1 plays a role in satiety.^44^

Finally, pathological processes as inflammation and endothelial dysfunction play a role in the etiology of cardiovascular events.^45^ ^46^ A previous study showed that higher adherence to a Mediterranean Style diet is associated with a lower concentration of biomarkers for inflammation and endothelial dysfunction; C reactive protein, interleukin-6, E-selectin and soluble intercellular cell adhesion molecule-1.^47^ Furthermore, the literature shows that olive oil, vegetables, cereals and nuts have antithrombotic and/or anticoagulant effects.^46^ Adherence to a Mediterranean Style diet is associated with lower levels of prothrombotic biomarkers, for example, fibrogen,^48^ which contributes to a lower cardiovascular risk as well.

Our study has several limitations. Although this is a prospective cohort study, causality of the associations cannot be assumed, since this study is of observational nature. Also the number of NODAT cases and the number of deaths is small. Furthermore, the FFQ was originally developed to examine protein intake in RTR. It was only validated by comparing the protein intake of the FFQ with the protein intake calculated by the Maroni Equation, using urinary urea excretion values.^19^ The Mediterranean Style diet of our RTR is not optimal and may further lower the risk of NODAT and all-cause mortality through a better adherence to the traditional Mediterranean diet. It is the first time the association between the Mediterranean Style diet and NODAT and all-cause mortality is investigated in RTR. Furthermore, strengths include a complete follow-up of the clinically relevant end points: NODAT and all-cause mortality.

In conclusion, our prospective cohort study suggests that higher adherence to a Mediterranean Style diet may prevent the development of NODAT and all-cause mortality in RTR. More attention is needed for the nutritional habits of RTR.
