# Supplementary material for: Mediterranean style diet is associated with low risk of new-onset diabetes after renal transplantation
Source: BMJ Open Diabetes Res Care. 2017 Jan 13;5(1):e000283. doi: 10.1136/bmjdrc-2016-000283 (PMC5253439; doi:10.1136/bmjdrc-2016-000283)
Supplement: supplementary table — Overview of the food items. [file bmjdrc-2016-000283supp_table.pdf]

Supplementary Table 1. Overview of the food items.

1) **Ratio mono-unsaturated:saturated fatty acids**

2) **Legumes, nuts and soy products**
Legumes
Soy products
Peanuts (and coated peanuts)
Nuts

3) **Cereals**
Rusk
Croissants
Bread rolls
Cereals
Pasta
Rice
Bread
Rye bread
Raisin bread

4) **Fruit**
Citrus fruit
Other fruit
Fruit in can/jar

5) **Vegetables**
Broccoli, cauliflower etc.
Sal, spinach, endive etc.
Mushrooms
Onion, pepper etc.
Raw vegetables
Vegetable juice

6) **Meat products**
Chicken, turkey
Beef: steak etc.
Beef: blind finch etc.
Pork: steak etc.
Pork: chop etc.
Pork: smoked sausage etc.
Lamb or mutton
Other meats such as goat/horse
Cooked liver
Hepatic / renal products
Sausages
Minced meat (all types)
Liver products
Ham etc.
Luncheon meat etc.
Bacon etc.
Unknown type of meat

7) **Dairy products**
Full milk
Semi-skimmed milk
Skimmed milk
Buttermilk
Unknown type of milk
20+ / 30+ cheese
40+ cheese
Ordinary cheese (48+)
Luxury fat cheese
Less fat cheese
Unknown type of cheese
Cheese used in hot meal
Cheese as in between
Full (fruit) yogurt
Semi-skimmed (fruit) yogurt
Skimmed (fruit) yogurt
Yogurt with probiotics
Full custard & pudding
Low-fat custard & pudding
Lean custard & pudding
Full (fruit) cream cheese
Semi-skimmed (fruit) cream cheese
Skimmed (fruit) cream cheese
Ice cream
Whipped cream
Other milk, such as horse and goat
Unknown dairy drink
Hot porridge
Normal milk in coffee
Coffee milk, coffee cream
Cream used in hot meals
Full fat milk beverages
Semi-skimmed fat milk beverages
Skimmed fat milk beverages
Breakfast drink
Butter
Low-fat butter
Beverages with probiotics
Cholesterol-lowering yoghurt drinks

8) **Fish**
Shellfish
Trout, mullet, plaice etc.
Salmon and other fatty fish
Herring
Fried fish
Unknown fish species

9) **Alcohol**
Beer
Wine
Cocktails
Spirits
